# Differential Effect of Omega-3 Fatty Acids on Platelet Inhibition by Antiplatelet Drugs In Vitro

**DOI:** 10.3390/ijms251810136

**Published:** 2024-09-21

**Authors:** Ioannis K. Koutsaliaris, Despoina Pantazi, Aikaterini N. Tsouka, Ourania Argyropoulou, Constantinos C. Tellis, Alexandros D. Tselepis

**Affiliations:** Atherothrombosis Research Centre/Laboratory of Biochemistry, Department of Chemistry, University of Ioannina, 45100 Ioannina, Greece; i.koutsaliaris@uoi.gr (I.K.K.); dpantazi@uoi.gr (D.P.); a.tsouka@uoi.gr (A.N.T.); rea.ioannina@yahoo.gr (O.A.); ktellis@uoi.gr (C.C.T.)

**Keywords:** antiplatelet drugs, DHA, EPA, omega-3 fatty acids, platelets, PUFAs

## Abstract

The omega-3 polyunsaturated fatty acids (PUFAs) Docosahexaenoic acid (DHA) and Eicosapentaenoic acid (EPA) exert multiple cardioprotective effects, influencing inflammation, platelet activation, endothelial function and lipid metabolism, besides their well-established triglyceride lowering properties. It is not uncommon for omega-3 PUFAs to be prescribed for hypertriglyceridemia, alongside antiplatelet therapy in cardiovascular disease (CVD) patients. In this regard, we studied the effect of EPA and DHA, in combination with antiplatelet drugs, in platelet aggregation and P-selectin and α_IIb_β_3_ membrane expression. The antiplatelet drugs aspirin and triflusal, inhibitors of cyclooxygenase-1 (COX-1); ticagrelor, an inhibitor of the receptor P2Y_12_; vorapaxar, an inhibitor of the PAR-1 receptor, were combined with DHA or EPA and evaluated against in vitro platelet aggregation induced by agonists arachidonic acid (AA), adenosine diphosphate (ADP) and TRAP-6. We further investigated procaspase-activating compound 1 (PAC-1) binding and P-selectin membrane expression in platelets stimulated with ADP and TRAP-6. Both DHA and EPA displayed a dose-dependent inhibitory effect on platelet aggregation induced by AA, ADP and TRAP-6. In platelet aggregation induced by AA, DHA significantly improved acetylsalicylic acid (ASA) and triflusal’s inhibitory activity, while EPA enhanced the inhibitory effect of ASA. In combination with EPA, ASA and ticagrelor expressed an increased inhibitory effect towards ADP-induced platelet activation. Both fatty acids could not improve the inhibitory effect of vorapaxar on AA- and ADP-induced platelet aggregation. In the presence of EPA, all antiplatelet drugs displayed a stronger inhibitory effect towards TRAP-6-induced platelet activation. Both omega-3 PUFAs inhibited the membrane expression of α_IIb_β_3_, though they had no effect on P-selectin expression induced by ADP or TRAP-6. The antiplatelet drugs exhibited heterogeneity regarding their effect on P-selectin and α_IIb_β_3_ membrane expression, while both omega-3 PUFAs inhibited the membrane expression of α_IIb_β_3_, though had no effect on P-selectin expression induced by ADP or TRAP-6. The combinatory effect of DHA and EPA with the antiplatelet drugs did not result in enhanced inhibitory activity compared to the sum of the individual effects of each component.

## 1. Introduction

Omega-3 (n-3) polyunsaturated fatty acids (PUFAs) are a group of essential, long-chain fatty acids that contain two or more cis double bonds, the last of which is positioned three carbon atoms away from the methyl terminal group [1,2]. Among omega-3 PUFAs, Eicosapentaenoic acid (EPA, 20:5n-3) and Docosahexaenoic acid (DHA, 22:6n-3), mainly found in vegetable oils, eggs, nuts and fatty fish, are the most functionally important and exert multiple cardioprotective effects [3,4]. Although a body of clinical and in vitro studies focused on the potential role of EPA and DHA in reducing the risk of cardiovascular disease (CVD) have conflicting outcomes, many large studies have shown a prospective link between omega-3 consumption and reduced cardiovascular (CV) risk [5,6,7,8]. There is well-documented evidence that consumption of EPA and/or DHA in pharmacological doses can aid in serum triglyceride (TG) reduction [1,9]. As a result, the American and European guidelines recommend the prescription of omega-3 PUFAs in patients with elevated TGs. Beyond their TG-lowering properties, the omega-3 PUFAs EPA and DHA display variable beneficial effects in many crucial aspects of atherosclerosis and thrombosis [10,11,12]. Clinical and in vitro studies show that these omega-3 PUFAs reduce plasma inflammatory markers, regulate lipoprotein metabolism, inhibit platelet activation and aggregation, inhibit lipoprotein and cell membrane oxidation, and protect against apoptosis and cell death in atherosclerosis [1]. Furthermore, their bioactive metabolites called specialized pro-resolving mediators (SPMs), such as resolvins, protectins and maresins, exert multiple cardioprotective effects and are well-established regulators of the resolution phase of inflammation [13]. A study with high significance, among a great number of clinical trials, The Reduction of Cardiovascular Events with EPA-Intervention Trial (REDUCE-IT) demonstrated that daily intake of 2 × 2 g Icosapent Ethyl (IPE), a highly purified ethyl ester of EPA, reduced cardiovascular events by 25% compared with placebo in statin-treated high-CV-risk patients [14]. Nevertheless, the clinical significance of omega-3 PUFAs consumption in primary or secondary CVD prevention needs further investigation.

Thrombus formation is the key pathophysiological factor in the development of ischemic events [15]. The complex mechanisms that characterize thrombus formation elicit the development of highly specialized antiplatelet and anticoagulant therapies for the prevention and treatment of CVD. These therapies potently inhibit thrombosis and cause less bleeding events [16,17,18,19].

Antiplatelet drugs that are used in daily clinical practice are the cyclooxygenase-1 (COX-1) inhibitors aspirin (acetylsalicylic acid; ASA) and triflusal, purinergic receptor P2Y_12_ antagonists (prasugrel, clopidogrel, ticagrelor), integrin receptor αIIb/β3 (glycoprotein IIb/IIIa; GPIIb/IIIa) antagonists (abciximab, eptifibatide) and the Protease-Activated Receptor-1 (PAR-1) antagonist vorapaxar [20].

ASA irreversibly inhibits COX-1, thus blocking the production of thromboxane A2 (TXA2) [21,22]. Even though administration of ASA can lead to gastrointestinal toxicity, aspirin remains the primary therapeutic option for CVD prevention, including acute events [23,24,25,26,27]. Triflusal has a similar-to-aspirin efficacy in preventing CV events and is used in clinical practice as a secondary prevention antiplatelet drug [28]. Even though triflusal and aspirin exhibit similar chemical and functional characteristics, triflusal administration is associated with lower-than-ASA bleeding risk [28].

The P2Y_12_ antagonists clopidogrel and prasugrel are prodrugs whose active metabolites are formed in vivo and bind irreversibly to the P2Y_12_ receptor, inhibiting adenosine diphosphate (ADP)-mediated platelet activation. By contrast, ticagrelor is administered in an active form and binds reversibly to the P2Y_12_ receptor, inhibiting ADP-mediated receptor activation [29]. Dual antiplatelet therapy (DAPT), which consists of aspirin and a P2Y_12_ antagonist, is an established therapy in daily clinical practice to treat patients with an acute coronary syndrome (ACS) [30].

Vorapaxar inhibits platelet activation via blocking the PAR-1 receptor. Vorapaxar is administrated in patients with stable atherosclerotic CVD who are receiving standard antiplatelet therapy and leads to the reduction of ischemic events or CV death. However, vorapaxar administration is associated with increased risk of moderate or severe bleeding, including intracranial hemorrhage [31].

CVD patients receiving DAPT or monotherapy with an antiplatelet drug may also exhibit high TG levels. These patients are advised to additionally receive IPE, or the combination of EPA with DHA, and/or to consume foods enriched in these PUFAs [9,32]. Therefore, the aim of the present study was to investigate the inhibitory effect of EPA and DHA, either alone or in combination with ASA, triflusal, ticagrelor or vorapaxar, on platelet activation induced by three different agonists, Arachidonic Acid (AA), ADP or the PAR-1 agonist TRAP-6 (thrombin receptor activating peptide 6), in vitro.

## 2. Results

### 2.1. Estimation of Antiplatelet Drug’s Effective Concentration That Inhibits by up to 30% Platelet Aggregation Induced by Various Agonists

In preliminary experiments, we performed platelet aggregation studies of untreated platelets using AA (300 µM), ADP (6 µM) and TRAP-6 (10 µM) as agonists. In these studies, AA (300 µM), ADP (6 µM) and TRAP-6 (10 µM) induced platelet aggregation by 82.47 ± 3.32, 94.90 ± 1.76 and 88.41 ± 4.45, respectively. Subsequently, we used a range of ASA, triflusal, ticagrelor and vorapaxar concentrations to estimate the doses that inhibit, by up to 30%, the above platelet aggregation induced by all three agonists. The purpose of these experiments was to identify the appropriate concentration of each antiplatelet drug that would enable the study of its inhibitory effect in combination with EPA or DHA and determine any possible additive or synergistic effect between the antiplatelet drugs and omega-3 PUFAs. We considered the synergistic effect between the antiplatelet drugs and omega-3 PUFAs as any combinatory effect greater than the sum of the individual effects displayed by each drug and omega-3 PUFA alone. To determine any possible synergy among the substances under investigation, we established a 30% inhibitory effect cutoff, which would allow us to detect significantly higher inhibitory levels.

**Figure 1:**
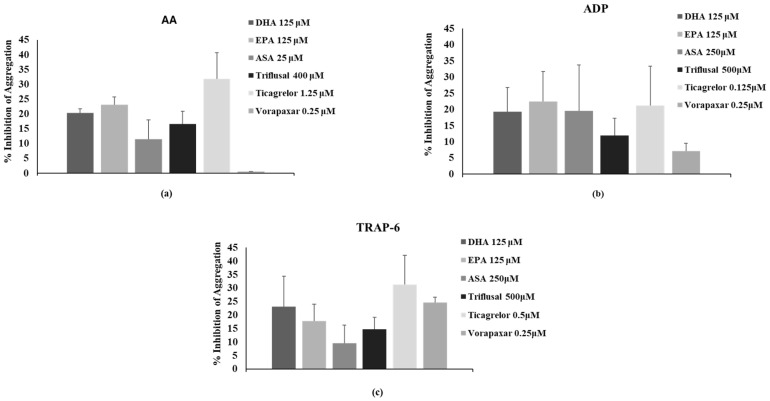
The omega-3 polyunsaturated fatty acid (PUFA) and antiplatelet drug concentrations that induce an up-to-30% inhibition of platelet aggregation induced by (**a**) Arachidonic acid (AA), (**b**) adenosine diphosphate (ADP) and (**c**) TRAP-6. The values represent the Mean ± SD from at least three different platelet preparations.

According to our results, ASA at concentrations of 25 μΜ, 250 μΜ and 250 μΜ inhibited platelet aggregation induced by AA, ADP and TRAP-6 by 11.49 ± 6.48%, 19.55 ± 14.16% and 9.56 ± 6.64%, respectively. ASA at 50 µM inhibited AA-induced platelet activation by 42.03 ± 9.89%; therefore, the concentration of 25 μΜ was utilized in further experiments. Furthermore, ASA at concentrations higher than 250 μΜ did not display any stronger inhibitory effect towards platelet aggregation induced by ADP and TRAP-6 (47.37 ± 11.40% and 38.14 ± 9.31%, respectively, at 500 μΜ ASA); therefore, the minimal ASA concentration of 250 μΜ, which induced the maximum inhibitory effect, was selected to be studied in combination with EPA or DHA, in the presence of the above two agonists. Triflusal at 400 μΜ inhibited by 16.62 ± 4.30% and at 800 µM by 68.58 ± 6.13% the AA-induced platelet aggregation; therefore, and in accordance with the above, the concentration of 400 μΜ was used in further experiments. Moreover, triflusal at 500 μΜ inhibited by 11.99 ± 5.24% and 14.70 ± 4.45% ADP- and TRAP-6-induced platelet aggregation and at 1000 µM by 34.71% and 36.50%, respectively. Consequently, the concentration of 500 μΜ was used in further experiments with the above two agonists. Ticagrelor at 1.25 μΜ inhibited by 31.81 ± 8.83% AA-induced platelet aggregation, whereas at 2.5 μΜ its inhibitory effect rose to 76.32 ± 17.56%. At 0.125 μΜ, ticagrelor inhibited by 21.27 ± 12.11% ADP-induced platelet aggregation, whereas at 0.250 μΜ an inhibitory effect of 62.51 ± 10.37% was observed. Therefore, the ticagrelor concentration of 0.125 μΜ was used in further experiments with ADP. Ticagrelor at 0.5 µM inhibited TRAP-6 induced platelet aggregation by 31.39 ± 10.75%, whereas at 1 µM an inhibitory effect of 73.75 ± 17.28% was displayed. Finally, vorapaxar at 2.5–5 μΜ displayed no inhibitory effect when AA or ADP were used as platelet agonists, while at 0.25 µM and 0.5 µM it inhibited TRAP-6-induced platelet aggregation by 24.65 ± 2.01% and 43.82 ± 17.51, respectively. Accordingly, the concentration of 0.25 µM was used for AA, ADP and TRAP-6. The above results regarding the effective concentrations that result in 30% inhibition are summarized in Figure 1.

### 2.2. Dose-Dependent Inhibitory Effect of DHA and EPA on Platelet Aggregation Induced by Various Agonists

**Figure 2:**
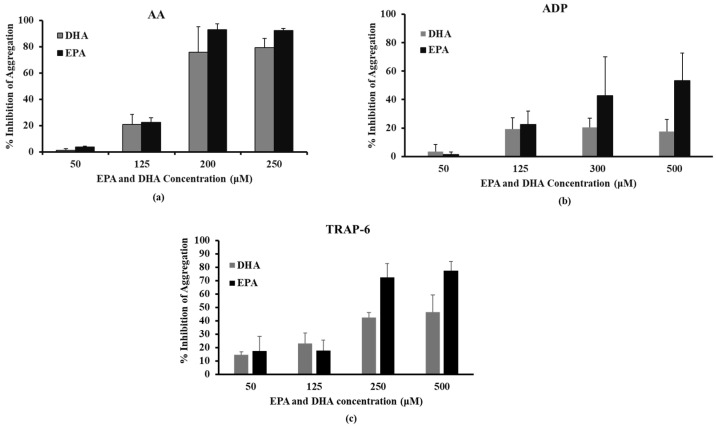
Dose-dependent effect of platelet aggregation in (**a**) AA (300 μΜ), (**b**) ADP (6 µM), (**c**) TRAP-6 (10 μΜ). Values represent Mean ± SD from at least three different platelet preparations.

The dose-dependent inhibitory effect of DHA and EPA on platelet aggregation induced by AA, ADP and TRAP-6 is shown in Figure 2. As previously described, we aimed to estimate the omega-3 PUFA concentrations that exhibit an up-to-30% inhibitory effect on platelet aggregation induced by AA (0.3 mM), ADP (6 µM) and TRAP-6 (10 µM). DHA and EPA at 125 μΜ inhibited AA-induced platelet aggregation by 20.38 ± 1.37% and 23.14 ± 2.60%, respectively (Figure 2a). Additionally, when ADP was used as an agonist, DHA and EPA at 125 μΜ inhibited platelet aggregation by 19.37 ± 7.43% and 22.46 ± 9.29%, respectively (Figure 2b). Finally, at 125 μΜ DHA and EPA caused 23.06 ± 11.41% and 17.87 ± 6.11% inhibition of platelet aggregation induced by TRAP-6, respectively (Figure 2c).

### 2.3. Effects of DHA or EPA Combinations with Antiplatelet Drugs on Platelet Aggregation Induced by AA

**Figure 3:**
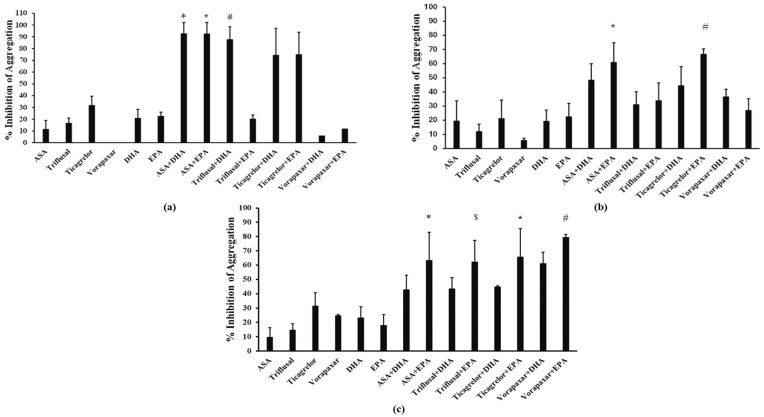
Bar graphs illustrating the % of inhibition by (**a**) Docosahexaenoic Acid (DHA) (125 µM), Eicosapentaenoic Acid (EPA) (125 µM), Acetylsalicylic Acid (ASA) (25 µM), triflusal (400 µM), ticagrelor (1.25 µM) and vorapaxar (0.25 µM) as well as the PUFA/antiplatelet drug combinations on platelet aggregation induced by AA (300 μΜ), * *p* < 0.001 compared with ASA and DHA; * *p* < 0.001 compared with ASA and EPA; # *p* < 0.001 compared with triflusal and DHA, (**b**) DHA (125 µM), EPA (125 µM), ASA (250 µM), triflusal (500 µM), ticagrelor (0.125 µM) and vorapaxar (0.25 µM) as well as the PUFA/antiplatelet drug combinations on platelet aggregation induced by ADP (6 μΜ), * *p* < 0.01 compared with ASA and EPA; # *p* < 0.01 compared with ticagrelor and EPA, (**c**) DHA (125 µM), EPA (125 µM), ASA (250 µM), triflusal (500 µM), ticagrelor (0.5 µM) and vorapaxar (0.25 µM) as well as the PUFA/antiplatelet drug combinations on platelet aggregation induced by TRAP-6 (10 μΜ), * *p* < 0.01 compared with ASA and EPA; $ *p* < 0.01 compared to triflusal and EPA; * *p* < 0.01 compared to ticagrelor and EPA; # *p* < 0.01 compared to vorapaxar and EPA. Values represent Mean ± SD from at least three different platelet preparations.

The combination of 25 µM ASA with 125 µM DHA or EPA displayed a 92.66 ± 9.52% and 92.59 ± 9.52% inhibition of AA-induced platelet aggregation, respectively, which was significantly higher (*p* < 0.005) compared with ASA, DHA or EPA alone (Figure 3a). Importantly, the combination of ASA with DHA or EPA displayed a higher inhibitory effect than the expected cumulative effect of their constituents (*p* < 0.05, compared to the cumulative inhibition of ASA + DHA and ASA + EPA, respectively), indicating that EPA and DHA have a priming effect on the antagonistic activity of ASA against AA (Table 1). The combination of 400 μΜ triflusal with 125 µM DHA inhibited by 89.12 ± 6.28% the AA-induced platelet aggregation, this being significantly higher (*p* < 0.005) compared with the combination of triflusal with 125 µM EPA (39.54 ± 9.50%) or with triflusal, DHA or EPA alone (Figure 3a). However, the inhibitory effects of the above combinations were similar to the expected cumulative effect of their constituents (Table 1). Furthermore, the combination of 1.25 μΜ ticagrelor with 125 µM of either DHA or EPA exhibited a 77.12 ± 23.13% and 79.03 ± 18.89% inhibition, respectively, both being stronger effects compared with ticagrelor, DHA or EPA alone (Figure 3a). The inhibitory effects of the above combinations were similar to the expected cumulative effect of their constituents (Table 1). Finally, no statistically significant inhibitory effect (<20%) was observed when 0.25 µM vorapaxar was combined with 125 µM of either DHA or EPA (Figure 3a).

### 2.4. Effects of Combinations of DHA or EPA with Antiplatelet Drugs on Platelet Aggregation Induced by ADP

The inhibitory effect of 250 µM ASA combined with 125 µM EPA on ADP-induced platelet aggregation was 61.92 ± 11.61%, being significantly higher (*p* < 0.01) compared with ASA or EPA alone. Importantly, the combination of ASA with EPA displayed a higher inhibitory effect than the expected cumulative effect of its constituents (*p* < 0.05, compared to the cumulative inhibition of ASA + EPA), implying that EPA has a priming effect on the antagonistic activity of ASA against ADP-induced platelet aggregation (Table 1). In contrast, the combination of 25 µM ASA with 125 µM DHA did not display a greater antiplatelet effect (47.40 ± 6.45%) compared with ASA, DHA or the cumulative inhibition of ASA and DHA (Figure 3b). The combination of 0.125 µM ticagrelor with 125 µM EPA exhibited a 67.40 ± 8.71% platelet inhibition, which is significantly stronger compared to ticagrelor or EPA alone (*p* < 0.01 for both comparisons). However, the effect of this combination was similar to the expected cumulative effect of its constituents (Table 1). The DHA and EPA combinations with 500 µM triflusal or vorapaxar did not display an improved inhibitory effect, compared with DHA, EPA, triflusal or vorapaxar alone (Figure 3b).

### 2.5. Effects of DHA or EPA Combinations with Antiplatelet Drugs on Human Platelet Aggregation Induced by TRAP-6

The inhibitory effect of 250 μΜ ASA in combination with 125 µM EPA or DHA towards TRAP-6-induced platelet aggregation was 63.21 ± 19.89% and 42.74 ± 10.21%, respectively. The ASA/EPA combination showed an enhanced inhibition of platelet aggregation compared with ASA or EPA alone (*p* < 0.05) (Figure 3c). The antiplatelet activity of 0.5 µM ticagrelor when combined with EPA was also increased to 67.71 ± 9.18% (*p* < 0.05 compared with ticagrelor or EPA alone). Similarly, the inhibitory effect of 500 μΜ triflusal combined with 125 µM DHA or EPA was 43.42 ± 7.80% and 62.24 ± 13.61%, respectively, EPA enhancing again the inhibitory activity of triflusal (*p* < 0.05 compared with triflusal or EPA alone) (Figure 3c). Finally, the combination of 0.25 µM vorapaxar with DHA or EPA inhibited TRAP-6-induced platelet aggregation by 60.14 ± 9.23% and 81.71 ± 3.01%, respectively. The combination of EPA with vorapaxar had an improved inhibitory effect (*p* < 0.05 compared with vorapaxar or EPA alone) (Figure 3c). Additionally, the vorapaxar and EPA combination displayed a higher inhibitory effect than the expected cumulative effect of its constituents (*p* < 0.05 compared to the cumulative inhibition of vorapaxar + EPA), suggesting that EPA has a priming effect on the antagonistic activity of vorapaxar against TRAP-6 (Table 1). The additive and experimental inhibitory effects of the above combinations are summarized in Table 1.

### 2.6. Effects of DHA or EPA Combinations with Antiplatelet Drugs on P-Selectin Membrane Expression and PAC-1 Binding

We further investigated the inhibitory effect of the omega-3 PUFAs and antiplatelet drugs, in combination or alone, on the conformational change of the integrin receptor α_IIb_β_3_ into its active form (PAC-1 binding) and the membrane expression of P-selectin induced by 50 μΜ ADP or 50 μΜ TRAP-6. Both DHA and EPA inhibited the PAC-1 binding induced by ADP (47.07 ± 27.67% and 52.46 ± 10.07, respectively) and TRAP-6 (38.84 ± 13.67% and 62.43 ± 25.25, respectively) (*p* < 0.05 compared with activated platelets), while they had no significant effect in the membrane expression of P-selectin induced by both agonists (Table 2 and Table 3). Among the antiplatelet drugs, only ticagrelor had an inhibitory effect on both PAC-1 binding induced by ADP and TRAP-6 (58.86 ± 11.36% and 50.56 ± 10.44, respectively) and P-selectin expression induced by both ADP and TRAP-6 (51.13 ± 30.21% and 31.00 ± 21.97%, respectively). ASA and triflusal had no statistically significant effect on both platelet activation parameters. Vorapaxar inhibited TRAP-6-induced PAC-1 binding and P-selectin expression by 41.26 ± 6.59% and 54.85 ± 12.13%, respectively. The combination of vorapaxar and EPA had a stronger inhibitory effect (93.35 ± 9.41%) on PAC-1 binding (*p* < 0.05 compared to vorapaxar or EPA alone). Finally, the combinations of DHA or EPA with the antiplatelet drugs ASA, ticagrelor and triflusal did not display any statistically significant synergistic inhibitory effect on the membrane expression of either α_IIb_β_3_ or P-selectin (Table 2 and Table 3).

## 3. Discussion

Omega-3 PUFAs exhibit antiplatelet activity through diverse pathways. In this study, both DHA and EPA display dose-dependent inhibition of platelet aggregation when activators of COX-1, P2Y_12_ and PAR-1 were utilized. The incubation time of 5 min for the aggregatory and 10 min for P-selectin expression and PAC-1 binding studies was utilized to evaluate the antiplatelet effect of the two omega-3 PUFAs and minimize the influence of the SPMs of DHA and EPA produced after their metabolism, as the incubation time is not sufficient for the metabolism of DHA or EPA to occur in a high extent. Accordingly, the antiplatelet activity demonstrated in this study is not attributed to the products of omega-3 PUFA metabolism but to the fatty acids themselves.

In this study, both DHA and EPA displayed similar inhibitory potency toward platelet aggregation when activators of COX-1, P2Y_12_ and PAR-1 were utilized. It is well established that omega-3 PUFAs are incorporated into the cell membrane, differentially affecting its structure, fluidity and signal transduction [11,33]. Additionally, PUFAs influence cholesterol and sphingolipid enriched lipid domains in the cell membrane, called lipid rafts, which regulate cell signaling, membrane fluidity and protein trafficking. DHA and EPA are preferentially incorporated in lipid raft domains, modify their molecular organization and alter their properties [34]. Through these mechanisms, DHA and EPA can influence a range of membrane receptors, preventing platelet activation and aggregation. Furthermore, omega-3 PUFAs compete with AA for the same COX enzymes; in this way, they suppress its metabolism to pro-aggregatory prostaglandins (PGs) and thromboxanes and inhibit COX-dependent platelet activation. Moreover, the shift of the arachidonic acid/EPA ratio influences the G protein-coupled (GPCR) purinergic receptor P2Y_12_ [35]. Distinct membrane interactions greatly influence those effects, depending on the content of the cell membrane in EPA or DHA, as each PUFA impacts cell membrane interactions in a different way.

It is not uncommon for patients prescribed DAPT or monotherapy with aspirin or clopidogrel to simultaneously receive prescription grade omega-3 PUFAs for TG lowering. To determine the possible influence of omega-3 PUFAs and routinely prescribed antiplatelet drugs on platelet activation, we investigated the effects of their combinations. In this regard, we used the concentration of all PUFAs and drugs that resulted in up to 30% inhibition to determine any possible priming effect of their combinatory use. Both DHA and EPA potentiate the antiplatelet activity of ASA when AA was used as a platelet activator. Additionally, DHA has a priming effect on triflusal in AA-induced platelet aggregation. Furthermore, the combinations of EPA with ASA and EPA with ticagrelor display enhanced antiaggregatory activity compared to the sum of the combination’s constituents alone. The differential influence of DHA and EPA on platelet membrane structure and function along with the action of antiplatelet drugs on platelet receptors results in a case-by-case priming effect on antiplatelet activity. Combinations of EPA with ASA, triflusal, ticagrelor and vorapaxar display synergistic inhibitory activity more potent than their respective DHA combinations in PAR-1-mediated platelet aggregation.

In addition to the aggregatory studies, we evaluated the membrane expression of P-selectin and the integrin receptor α_IIb_β_3_ to determine the effect of DHA, EPA and their combinations with antiplatelet drugs on platelet secretion. Activated platelets express P-selectin, aiding the recruitment of monocytes, leukocytes and platelet aggregation, while the α_IIb_β_3_ receptor transits to its active state and mediates adhesion, platelet aggregation and thrombus formation [36,37]. DHA and EPA displayed an inhibitory effect on PAC-1 binding when ADP or TRAP-6 were used as agonists. The omega-3 PUFAs did not produce a priming effect in combination with antiplatelet drugs, but only the cumulative inhibitory effect of their components.

To summarize, the combinatory use of omega-3 PUFAs with antiplatelet drugs resulted in an enhanced antiplatelet effect, compared to the use of antiplatelet drugs alone, across all agonists that were utilized in this study. Moreover, the combination of ASA with DHA or EPA, as well as the ticagrelor and DHA combination, displayed a synergistic effect on AA-induced platelet aggregation. Likewise, the vorapaxar and EPA combination had a synergistic effect on platelet activation induced by TRAP-6. The omega-3 PUFAs did not significantly affect the activity of the antiplatelet drugs on P-selectin expression and PAC-1 binding, except in the case of vorapaxar, which exhibited an enhanced inhibitory effect on PAC-1 binding, induced by TRAP-6, when combined with EPA.

Overall, the aim of the present study is to enrich and clarify the current bibliography regarding the effect of omega-3 PUFAs in combination with the antiplatelet drugs used in clinical practice. However, this study is limited due to the in vitro nature of the experiments, meaning that the current results cannot be directly extrapolated to an in vivo study or clinical trial. The exploration of the molecular mechanisms underlying the effects presented in this study is the step that should follow these results. Although several studies attempt to shed light on the effects of omega-3 PUFAs on platelet functionality and drug response, in patients receiving monotherapy or DAPT, further studies are required to determine the cardiovascular benefits resulting from co-administration of PUFAs with antiplatelet therapy.

## 4. Materials and Methods

### 4.1. Chemicals and Reagents

DHA (FD180756) and EPA (FE22647) carboxylic acids were purchased by Biosynth Ltd. (Compton, UK), and ASA (10351.06.01) from Egicalm Galinos Company (Athens, Greece), triflusal was kindly provided from Galenica Company (Athens, Greece), ticagrelor was kindly provided by AstraZeneca (Gothenburg, Sweden), and vorapaxar (SCH 530348) from Axon MedChem (Groningen, The Netherlands). Arachidonic Acid (10931) was purchased from Sigma (St. Louis, MO, USA), ADP (P/N 384) and from Chrono-log (Havertown, PA, USA), and TRAP-6 (4017752) from Bachem (Bubendorf, Switzerland). The fluorescently labeled antibodies anti-PAC-1-FITC (340507), anti-CD62P-PE (348107), anti-CD61-PerCP (340506) were purchased from Becton- Dickinson (San Jose, CA, USA).

### 4.2. Platelet Aggregation Studies

Platelet Rich Plasma (PRP) was prepared from citrated blood of apparently healthy volunteers, as we have previously described [38,39,40,41,42,43], and platelets were adjusted to 250 × 10^6^/mL with homologous Platelet Poor Plasma (PPP). The study protocol was approved by the institutional ethics committee, and all participants gave their written informed consent before blood was drawn. Preliminary studies were performed to determine the minimum and maximum effective concentration of each PUFA and antiplatelet agent. For this purpose, platelets in 0.5 mL PRP aliquots were pre-incubated with EPA or DHA diluted in DMSO, or the antiplatelet drugs ASA, triflusal, ticagrelor or vorapaxar, or the combination of EPA or DHA with each one of the above drugs at 37 °C for 5 min. Platelet activation was induced by 300 µM AA in EtOH, 6 µM ADP in saline solution or 10 µM TRAP-6 in saline solution at 37 °C, in a Chronolog Lumi-Aggregometer, under continuous stirring at 1200 rpm, and the platelet aggregation curves were monitored for 5 min after the addition of each platelet agonist using the AggroLink (ver. 8.0)software package [38,39,40,41,42,43]. The final DMSO and/or EtOH concentration in each assay did not exceed 1% (*v*/*v*). All aggregation assays were conducted within 5 h after blood sample collection.

### 4.3. P-Selectin Membrane Expression and PAC-1 Binding

PRP was prepared as previously described, and platelets (250 × 10^6^/mL) were incubated with 1000 µM DHA or 1000 µM EPA and/or 25 µM ASA, 500 µM triflusal, 0.5 µM ticagrelor, 0.25 µM vorapaxar for 10 min at 37 °C [38]. In experiments where the PUFA/antiplatelet drug combinations were used, both agents were added simultaneously for a total incubation time of 10 min. Subsequently, 50 μΜ ADP or 50 μΜ TRAP-6, or their vehicle (saline solution), was added for 5 min at 37 °C, without stirring. Then, platelets were incubated with anti-PAC-1-FITC and anti-CD62P-PE and diluted with PBS 1:5 *v*/*v* (10 mM, pH = 7.4) for 20 min, in the dark, at room temperature. The surface expressions of P-selectin and the PAC-1 binding on activated platelets were studied in a FACSCalibur flow cytometer (Becton-Dickinson, San Jose, CA, USA) using the corresponding fluorescently labeled monoclonal antibodies anti-CD62P-PE and anti-PAC-1-FITC [40,44]. Platelets were gated according to staining for the platelet’s specific anti-CD61-PerCP. The gated events were further analyzed in histograms using the mean fluorescence intensity (MFI). Flow cytometry results are presented as the MFI of the activated sample minus the MFI of the resting sample, as we have previously described [43].

### 4.4. Statistics

All data are expressed as mean ± standard deviation (SD) from at least 3 independent experiments. Statistical Package for the Social Sciences (SPSS Inc., Chicago, IL, USA), v25 was used for statistical analysis. Results are expressed as mean ± SD. Mean values were compared by Student’s t test with significance defined at a value of *p* < 0.05.

## Figures and Tables

**Table 1:** The mean ± SD values of the sum of inhibitory effects and experimental inhibitory effects of the PUFA/antiplatelet drug combinations on platelet aggregation induced by Arachidonic Acid (AA), Adenosine Diphosphate (ADP) and TRAP-6. * *p* < 0.05 compared to the sum of Acetylsalicylic Acid (ASA) + Docosahexaenoic Acid (DHA) inhibitory effects, ** *p* < 0.05 compared to the sum of ASA + Eicosapentaenoic Acid (EPA) inhibitory effects, *** *p* < 0.05 compared to the sum of triflusal + DHA inhibitory effects, $ *p* < 0.05 compared to the sum of vorapaxar + EPA inhibitory effects. The values represent the Mean ± SD from at least three different platelet preparations.

	AA		ADP		TRAP-6	
Combinations	Sum of Inhibitory Effects (%)	Experimental Inhibition (%)	Sum of Inhibitory Effects (%)	Experimental Inhibition (%)	Sum of Inhibitory Effects (%)	Experimental Inhibition (%)
ASA + DHA	31.87 ± 7.78	92.66 ± 9.52 *	38.92 ± 21.89	47.40 ± 6.45	32.62 ± 18.05	42.74 ± 10.21
ASA + EPA	34.63 ± 9.08	92.59 ± 7.04 **	42.01 ± 23.45	61.92 ± 11.61	27.43 ± 12.75	63.21 ± 19.89
Triflusal + DHA	37.00 ± 5.60	89.10 ± 6.20 ***	31.36 ± 12.67	31.07 ± 8.98	37.76 ± 15.86	43.42 ± 7.80
Triflusal + EPA	39.76 ± 6.90	39.50 ± 9.50	34.45 ± 14.53	33.89 ± 12.47	32.57 ± 10.56	62.24 ± 13.61
Ticagrelor + DHA	52.19 ± 10.13	77.12 ± 18.62	40.46 ± 19.54	45.72 ± 12.74	54.45 ± 22.16	44.76 ± 2.30
Ticagrelor + EPA	54.95 ± 11.16	79.03 ± 15.08	43.73 ± 21.40	67.40 ± 8.71	49.26 ± 16.86	67.71 ± 9.18
Vorapaxar + DHA	20.38 ± 1.30	<20%	26.51 ± 9.79	38.83 ± 7.15	47.71 ± 13.42	60.14 ± 9.23
Vorapaxar + EPA	23.14 ± 2.60	<20%	29.60 ± 11.62	27.38 ± 9.08	42.52 ± 8.12	81.71 ± 3.01 ^$^

**Table 2:** Mean fluorescence intensity values of P-selectin and integrin α_IIb_β_3_ membrane expression after ADP-induced platelet activation. Values represent Mean ± SD from at least three different platelet preparations.

	Membrane Expression of P-Selectin (CD62-PE)	Membrane Expression of α_IIb_β_3_ (PAC-1-FITC)
	Inhibition (%)	Inhibition (%)
DHA 1000 µM	3.38 ± 4.78	47.07 ± 27.67
EPA 1000 µM	6.49 ± 9.17	52.46 ± 10.07
ASA 25 µM	12.8 ± 18.11	14.57 ± 20.61
Tic 0.5 µM	51.13 ± 30.21	58.86 ± 11.36
Vor 0.25 µM	0.21 ± 0.30	15.67 ± 14.15
Triflusal 500 µM	0.00 ± 0.00	18.21 ± 25.76
ASA + DHA	31.00 ± 43.84	51.21 ± 49.20
ASA + EPA	14.50 ± 20.51	49.45 ± 10.54
Tic + DHA	42.78 ± 46.00	92.27 ± 7.79
Tic + EPA	35.22 ± 33.81	80.14 ± 1.41
Trifl + DHA	15.63 ± 20.32	63.84 ± 18.61
Trifl + EPA	3.94 ± 4.33	48.38 ± 1.95
Vor + DHA	7.46 ± 10.50	52.73 ± 7.74
Vor + EPA	21.4 ± 8.66	68.38 ± 5.12

**Table 3:** Mean fluorescence intensity values of P-selectin and integrin α_IIb_β_3_ membrane expression after TRAP-6-induced platelet activation. Values represent Mean ± SD from at least three different platelet preparations.

	Membrane Expression of P-Selectin (CD62-PE)	Membrane Expression of α_IIb_β_3_ (PAC-1-FITC)
	Inhibition (%)	Inhibition (%)
DHA 1000 µM	6.18 ± 5.74	38.84 ± 13.67
EPA 1000 µM	16.34 ± 14.15	62.43 ± 25.25
ASA 25 µM	0.00 ± 0.00	0.00 ± 0.00
Tic 0.5 µM	31.00 ± 21.97	50.56 ± 10.44
Vor 0.25 µM	41.26 ± 6.59	54.85 ± 12.13
Triflusal 500 µM	4.11 ± 5.82	0.00 ± 0.00
ASA + DHA	14.34 ± 8.04	22.17 ± 4.48
ASA + EPA	33.57 ± 9.08	33.83 ± 5.41
Tic + DHA	44.35 ± 33.60	81.51 ± 11.57
Tic + EPA	67.29 ± 9.03	78.86 ± 14.29
Trifl + DHA	38.29 ± 16.75	35.38 ± 4.53
Trifl + EPA	23.95 ± 9.08	26.89 ± 2.67
Vor + DHA	68.56 ± 11.24	85.13 ± 23.67
Vor + EPA	66.44 ± 14.81	93.35 ± 9.41

## Data Availability

All data is located at Atherothrombosis Research Center and will be available upon request from the corresponding author Alexandros D. Tselepis.
